# Invasive Fungal Diseases of Combat Wounds: Burden, Epidemiology, and Mycology

**DOI:** 10.1007/s11046-024-00908-4

**Published:** 2024-11-21

**Authors:** Ashleigh Roberds, Alexander G. Bobrov, Riina Rautemaa-Richardson, Thomas J. Walsh

**Affiliations:** 1 Wound Infections Department, Bacterial Diseases Branch, Center for Infectious Diseases Research, Walter Reed Army Institute of Research, Silver Spring, MD USA; 2 Division of Evolution, Infection and Genomics, Faculty of Biology, Medicine and Health, University of Manchester, Manchester, UK; 3 Department of Infectious Diseases, Mycology Reference Centre Manchester, ECMM Centre of Excellence, Manchester Academic Health Science Centre, Wythenshawe Hospital, Manchester University NHS Foundation Trust, Manchester, UK; 4 National Health Services, Mycology Reference Centre Manchester, Manchester, UK; 5 Center for Innovative Therapeutics and Diagnostics, Richmond, VA USA; 6 Departments of Medicine and Microbiology & Immunology, University of Maryland School of Medicine, Baltimore, MD USA

**Keywords:** Invasive fungal infections, Military medicine, Wound infections, Mucorales, Combat wounds

## Abstract

During the last two decades, wound invasive fungal diseases (WIFDs) have reemerged as important causes of mortality and morbidity in military personnel and civilian casualties in war areas. Historically, mycotic infections acquired in combat operations during Vietnam War and were associated with burn wounds. Modern combat related WIFDs are almost exclusively associated with severe traumatic events which encompass blast exposure as the primary mechanism of injury and subsequent extremity amputation and extensive blood loss. Such infections often lead to deep tissue necrosis, long hospitalizations, extensive surgeries, and more severe amputation. Studies of combat related WIFDs among U.S. military personnel in Operation Enduring Freedom (Afghanistan) demonstrated incidence rates of approximately 7% and crude mortality of 8.5%. WIFDs were also seen in U.K. military personnel returning from Afghanistan and are common in the current Ukraine and Gaza conflicts. *Mucorales*, *Aspergillus* and *Fusarium* species are the predominant causes of WIFDs. These molds are opportunistic pathogens which thrive in patients with immune system imbalances following traumatic injury. They are ubiquitous environmental fungi found in a variety of soils but there are significant regional differences depending on the local soil type, vegetation, and climate. The management of WIFDs is complicated by the limited efficacy of current antifungals on many of these environmental species and by emerging antifungal resistance globally. This review provides an overview of the global burden, epidemiology, and clinical features of combat-related fungal infections with the aim to provide a better understanding of the threat posed for wounded Service Members and civilians.

## Introduction

Combat environments present unique challenges for healthcare providers in managing both fungal and bacterial infections. Explosives, including mines, artillery shells, missiles, drones, and improvised explosive devices, cause severe polytraumatic injuries, compromising immune systems and creating a haven for both bacterial and fungal pathogens. While bacterial infections remain more common than fungal infections [1–3], fungal infections pose a growing threat due to their ability to cause aggressive invasive and angio-invasive infections. Initial contamination of combat wounds is usually managed with topical disinfectants, therapeutics, and antibiotics in the first echelons of care. However, persistent wound infections are often hospital-acquired and caused by multidrug-resistant (MDR) strains of *Pseudomonas aeruginosa*, *Klebsiella pneumoniae, Staphylococcus aureus,* and *Acinetobacter baumannii,* leading to localized cellulitis, abscess formation, bone or systemic infection if missed and left untreated [4, 5]. In most cases, these bacterial infections are well managed with a combination of surgery and targeted antibiotics [6]. In contrast, infections caused by environmental invasive molds like *Mucorales*, *Aspergillus*, and *Fusarium* species often present with more insidious clinical manifestations and require a higher index of suspicion for diagnosis and targeted diagnostics [7, 8]. Wound invasive fungal diseases (WIFDs) can be challenging to manage due to limited treatment options and the inherent resistance of many environmental molds to antifungals. This necessitates multiple surgeries and extended courses of antifungal therapy. In recent decades, the resurgence of WIFDs has posed a signification challenge in military healthcare systems worldwide, impacting both military personnel and civilian casualties. Despite advancements in combat medicine improving survival rates for traumatic injuries, the rise in WIFDs creates new challenges and limitations for healthcare providers and combat operations.

## History and Epidemiology of WIFDs

Throughout history, WIFDs have been a persistent concern in military operations, with notable occurrences documented in conflicts such as the Vietnam War. Table 1 summarizes WIFD data from some recent conflicts. In the wars of the early and mid-twentieth century, incidence and prevalence was wildly unknown often due to the limitation in medical documentation and diagnostic capabilities. These wars, however, involved military tactics and combat environments ripe for fungal disease. Specifically, prolonged environmental exposures associated with trench warfare, as well as general unsanitary/crowded conditions were contributing factors. In the Vietnam War, environmental conditions such as heat and humidity, and combat injuries such as burns and large wounds created ideal conditions for fungal growth and infection. In one study conducted by the U.S. Army Institute of Surgical Research (USAISR), 30 burn patients were infected with Phycomycetes (likely *Mucorales* spp.) and *Aspergillus* spp., of which nine patients died from fungal invasion and seven casualties required amputations to eliminate disease [9].

**Table 1:** Conflicts and Wound Invasive Fungal Diseases

Conflict	Invasive Fungal Wound Infections	Reference
Vietnam War	Burn patients 42.2% were colonized with yeast and fungi *Candida albicans*, Phycomycetes (Mucormycetes), *Aspergillus* sp. 4% were invasive fungal infections – all *Mucorales* spp. (histology)	[15]
Operation Enduring Freedom (Afghanistan)	Associated with: Blast exposure Dismounted (foot patrol) blast injuries in Southern Afghanistan Ground-emplaced improvised explosive devices (IEDs) Above the knee amputations Incident rates of approximately 7% Crude Mortality rate of 8.5% *Mucorales* species (*Saksenaea*, *Apophysomyces*, *Actinomucor, Rhizopus, and Mucor* species), *Aspergillus* species (*Aspergillus flavus* and *Aspergillus terreus)*, and *Fusarium* species	[11]; [10, 16] [17]
Operation Iraqi Freedom	• Invasive mucormycosis, due to *Actinomucor elegans* • *Aspergillus *(n = 4), *Bipolaris* (n = 2), and 1 each *Mucor* and *Absidia*	[18, 19]
Israeli conflicts in South Lebanon	2.6% of injuries caused by cluster munitions had fungal inoculations	[12]
Israeli conflicts in Gaza	WIFDs in 11 deployed Israeli Defense Force (IDF) service members	[13, 20]
Ukraine conflict	WIFDs in patients transferred to European medical centers (molds and yeasts including *Candida auris,* data preliminary and incomplete)	Personal correspondence (TW and RR-R)

In contemporary warfare and global conflict, there was a resurgence of WIFDs seen in Afghanistan [10, 11], Ukraine (personal correspondence, TW and RR-R), and Israeli conflicts in South Lebanon [12] and Gaza [13]. These conflicts have provided fertile ground for WIFDs associated with severe polytraumatic injuries [14] in deployed settings.

Studies conducted among U.S. military personnel in Afghanistan reported WIFD incidence rates of approximately 7%, with crude mortality rates of 8.5% (attributable rates were not determined) [10, 16], indicating the significant burden of WIFDs in combat environments. Similarly, the UK documented gross contamination with environmental molds amongst Service Members injured in the “Green Zone” or large agricultural area with lush vegetation, in Afghanistan [11]. Combat injuries of modern warfare are almost exclusively associated with blast exposures [17], which not only cause traumatic injuries that disrupt the body’s natural skin barriers, including burns and barotrauma, but also were shown to induce a temporary state of immune paralysis [21–23]. Consequently, during the Israeli conflict in South Lebanon, 2.6% of injuries caused by cluster munitions had fungal inoculations [12] and in Afghanistan, 96% of casualties who developed WIFDs had injuries caused by explosion [10, 11, 14, 17]. From 2004 to 2007, the most common cause of death amongst a cohort of U.S. military burn casualties was infection [24], and among 36 burn-associated deaths caused by infection, 20 (46%) were attributed to bacteria and 10 (28%) to fungi, especially *Mucorales* and *Aspergillus*.

Recently, there was a report of a cluster of severe WIFDs in 11 Israeli Defense Force (IDF) service members deployed to the conflict in Gaza [13, 20]. One of these IDF fighters died directly from his fungal infection. Others are still being treated. Reports of combat wounds being contaminated by water and soil polluted with organic waste and sewage on the battlefield are consistent with the acquisition of these WIFDs. By comparison, there is a dearth of reports on WIFDs from the more recent conflicts in Ukraine and Gaza. Initial reports of life-threatening antimicrobial resistant bacterial infections in patients sustaining combat injuries in Ukraine do not include fungal infections [25]. Whether this represents a true absence of WIFDs or limited diagnostic information, is uncertain. Nonetheless, the fertile soil of Ukrainian battlefields would suggest an ecosystem that would support potentially pathogenic fungi. Also, during their typically very long hospital stay, these patients are at risk for healthcare-associated fungal infections with both molds and yeasts (including *Candida auris*) throughout their journey.

In addition to their occurrence in military settings, WIFDs are also observed in civilians who have experienced traumatic events. Trauma-related WIFDs can arise for a variety of incidents beyond military activity, including motor vehicle accidents, natural disasters, and industrial accidents [26, 27]. In these scenarios, traumatic injuries disrupt the body’s natural mechanical barriers by creating entry points for fungal pathogens to invade and establish infections. Similar to military personnel, civilians affected by trauma-related WIFDs may experience a range of clinical manifestations, including localized wound infections and deep tissue necrosis [28, 29]. Despite the differences in setting and populations, trauma-related WIFDs represent a significant public and global health concern.

## Microbiology and Diagnostics

Understanding the microbiology of fungal pathogens is crucial for effective management of WIFDs in trauma settings. Angioinvasive filamentous fungi such as *Mucorales*, *Aspergillus*, and *Fusarium* species are commonly implicated in WIFDs among military personnel [30, 31]. While *Aspergillus flavus* and *Aspergillus terreus* were more predominant for Aspergillus, more specifically within the order Mucorales, *Saksenaea*, *Apophysomyces*, *Actinomucor, Rhizopus*, and *Mucor* species predominate and are the leading causes of post-traumatic mucormycosis [28, 31]. Fungal detection should prioritize molecular methods whenever possible. Culturing techniques can be unreliable for molds due to the fragility of their hyphae, which are sensitive to disruption during sample processing. Furthermore, certain fungal species, like *Saksenaea* and *Apophysomyces*, are notorious for their difficulty in forming sporangiospores in standard culture media. This limitation can lead to underdiagnosis using traditional microbiological methods [31, 32]. Mucormycosis is a highly aggressive and invasive disease where the fungi frequently adapt to diverse environmental conditions, develop resistance to common antifungal agents, and cause significant tissue necrosis [28]. Inoculation and colonization can occur from either the sporangiospore, as observed in sinopulmonary disease, or from environmental germinated sporangiospores/hyphae, where emerging evidence suggests hyphal elements in the form of soil-based mycelia are the principal infectious form in traumatic wounds [28, 32]. Notably, hyphae of *Rhizopus arrhizus* and *Lichtheimia corymbifera arrhizus* were 10–16 times more virulent than sporangiospores in a *Galleria melonella* model and developed sustained wound infection in a combat-relevant murine model of wound mucormycosis [32, 33]. These mycelia are also likely to be inoculated in biofilms on particles of soil, glass, wood, stone, and metal into traumatized tissue. The effect of severe trauma may create an immune paralysis characterized by cytokine dysregulation and impaired phagocytic function [28, 33].

To further complicate diagnostics and treatment, WIFDs are commonly associated with co-infections of bacterial pathogens. Most common bacteria isolated from wounds of patients with WIFDs were *Enterobacter* spp. (45%), *Escherichia coli* (23%), *Pseudomonas aeruginosa* (20%) and *Acinetobacter* spp. (20%) [30]. Co-infection of multiple fungal pathogens is also common; combat WIFDs wound cultures growing fungi in the order Mucorales present with a second non-Mucorales fungus 30% of the time [34]. Additional studies suggest that co-infection of two or more pathogens occurs in 27%-67% of all WIFDs [27].

Early diagnosis of WIFDs is of paramount importance for initiation of wound debridement and timely initiation of antifungal therapy. Prolonged field exposure without adequate mycological diagnostics before evacuation may increase the risk of progressive WIFD. Rodriguez and colleagues reported that a positive culture swab for a medically important filamentous fungus from a traumatized wound in the absence of necrosis does not warrant initiation of antifungal therapy [14]. However, by the time overt necrosis is present, an invasive fungal disease is well established, thus warranting thorough debridement and prompt administration of antifungal therapy.

The Joint Trauma System Clinical Practice Guideline (JTS CPG) recommends obtaining resected tissue from the necrotic wound from the operation theater for culture and histology. Histology is particularly useful as it can confirm the suspicion of fungal infection rapidly. Also, the fungal burden in these infections is often high which results in reasonable sensitivity. Staining with periodic acid-Schiff and Gomori methenamine silver is recommended to ease detection of fungi [34]. When available, pan-fungal PCR may further increase sensitivity of detection and provide some quantification and identification to species level, which can guide antifungal treatment. The sensitivity of pan-fungal PCR is increased when performed on tissue with evidence of angioinvasion.

## Clinical Manifestations and Correlation with Outcomes

WIFDs present with a diverse range of clinical manifestations and outcomes, depending on the type of fungus involved, the mechanism of injury, the severity of infection, and the timeliness of intervention [14, 34]. Common clinical presentations of post-traumatic WIFDs include deep tissue necrosis and increased systemic inflammatory responses manifested by high fever and leukocytosis [10]. In the most severe cases, untreated or inadequately managed WIFDs can progress rapidly, leading to septicemia, multi-organ failure, and death [28]. However, with prompt diagnosis and appropriate treatment, favorable outcomes are achievable, especially in cases of localized infections or early-stage systemic disease [31, 34]. In cases with persistent primary infections and secondary fungal invasion, wound management is critical for good outcomes but it is complicated and requires intensive medical and surgical interventions [35].

Cases of WIFDs are significantly associated high injury severity scores including extensive blood loss and lower extremity amputations, exacerbating the requirement for repeated surgical interventions [14]. In one study, the median number of surgical debridements required for proven WIFD cases was 17, and 19% of cases required higher-level amputations [36]. The surgical and medical interventions required to treat WIFDs often demand extensive resources and expertise, placing strain on military healthcare systems already stretched thin in combat environments. Furthermore, WIFDs may result in long-term disabilities, which can have profound implications for the quality of life and functional outcomes of affected individuals both during and after their military service.

## Treatment Challenges

Treating WIFDs in military settings is complicated and challenging, primarily due to emerging antifungal drug resistance and the limitations of existing treatment modalities [34]. The effectiveness of antifungal agents may be compromised by resistant strains, requiring alternative therapeutic approaches. U.S. Military JTS CPG recommends Dakin’s solution for local irrigation and requires dual systemic antifungal therapy, which includes liposomal amphotericin B (LAmB) and a triazole agent, most commonly voriconazole [34]. However, these agents have dose-limiting toxicity, and global antifungal resistance is emerging at rapid rates [37], highlighting the urgent need for development of new antifungal agents [38]. Mucorales species have elevated minimum inhibitory concentration (MIC) to AmB, while *Aspergillus* spp. are increasingly detected with triazole resistance and complete resistance to voriconazole [32, 37, 39]. *Aspergillus terreus* strains are also resistant to LAmB [30] and *Fusarium* spp. may be completely resistant to all currently licensed antifungal therapy [40], threatening military and civilian antifungal treatment strategies. Performing MIC assays with hyphal elements should be considered, as recent evidence suggests hyphal elements exhibit significantly higher resistance to antifungals compared to sporangiospores [32, 41]. Emergence of global antifungal resistance may be fueled by poor antimicrobial stewardship, to include but not limited to the overuse of broad-spectrum classes of antifungal agents, particularly the triazoles, as environmental/agricultural fungicides [42], as well as genetic mutations in fungal pathogens.

## Conclusions

WIFDs represent a significant and evolving challenge in military healthcare, with implications for increased morbidity, mortality, and operational effectiveness. Understanding the burden of WIFDs in this setting requires a multifaceted approach that encompasses epidemiological surveillance of antifungal resistance patterns, updated clinical management strategies, and advanced research into novel treatment modalities. Healthcare providers in diverse settings managing combat wounds must remain vigilant for both bacterial and fungal infections, implementing timely diagnostics and targeted treatment strategies to optimize outcomes and minimize the burden of infectious complications on military personnel. By addressing the challenges posed by WIFDs comprehensively, healthcare providers and military leaders can better safeguard the health and well-being of Service Members and civilians in war zones in challenging operational environments, ultimately enhancing mission success and ensuring the readiness of military forces worldwide.
